# Livestock–wildlife interactions: key aspects for reconnecting animal production and wildlife conservation

**DOI:** 10.1093/af/vfad069

**Published:** 2024-02-14

**Authors:** Alexandra Cravino, Alberto Perelló, Alejandro Brazeiro

**Affiliations:** Grupo Biodiversidad y Ecología de la Conservación, Instituto de Ecología y Ciencias Ambientales, Facultad de Ciencias, Universidad de la República. Montevideo, Uruguay; SabioTec Spin-off S.L. Edificio Incubadora de empresas UCLM. Camino de Moledores, s/n 13071 Ciudad Real, Spain; Grupo Biodiversidad y Ecología de la Conservación, Instituto de Ecología y Ciencias Ambientales, Facultad de Ciencias, Universidad de la República. Montevideo, Uruguay

**Keywords:** habitat heterogeneity, livestock, stocking rate, wildlife, win-win outcomes

**Implications**

- Human population growth has brought an increase in food, water, and land demands, as a result of which livestock production is increasing, with significant consequences for wildlife.
- Livestock production negatively impacts wildlife when it implies completely substituting native ecosystems for pasturelands; when it occurs on native grasslands or even partially modified savannas, the impacts on wildlife are usually minor but highly dependent on stocking rate and management.
- Livestock production can reduce the abundance and alter the behavior of some wild species and even lead to their extinction at a local level by changing the vegetation structure and promoting a high presence of dogs and humans. The new environmental conditions that livestock generate could, nevertheless, favor some species.
- To benefit wildlife and sustainable production, livestock breeders should adjust stocking rates to intermediate levels to avoid severe soil and vegetation degradation and should opportunely rotate the herd between paddocks to generate heterogeneous landscapes.
- Conservationists and rangeland managers should promote dialogue among livestock breeders and scientists to find sustainable alternatives to favor wildlife, such as developing market distinctions and governmental support for good practices, with win-win outcomes.

## Background

**Figure 1.**
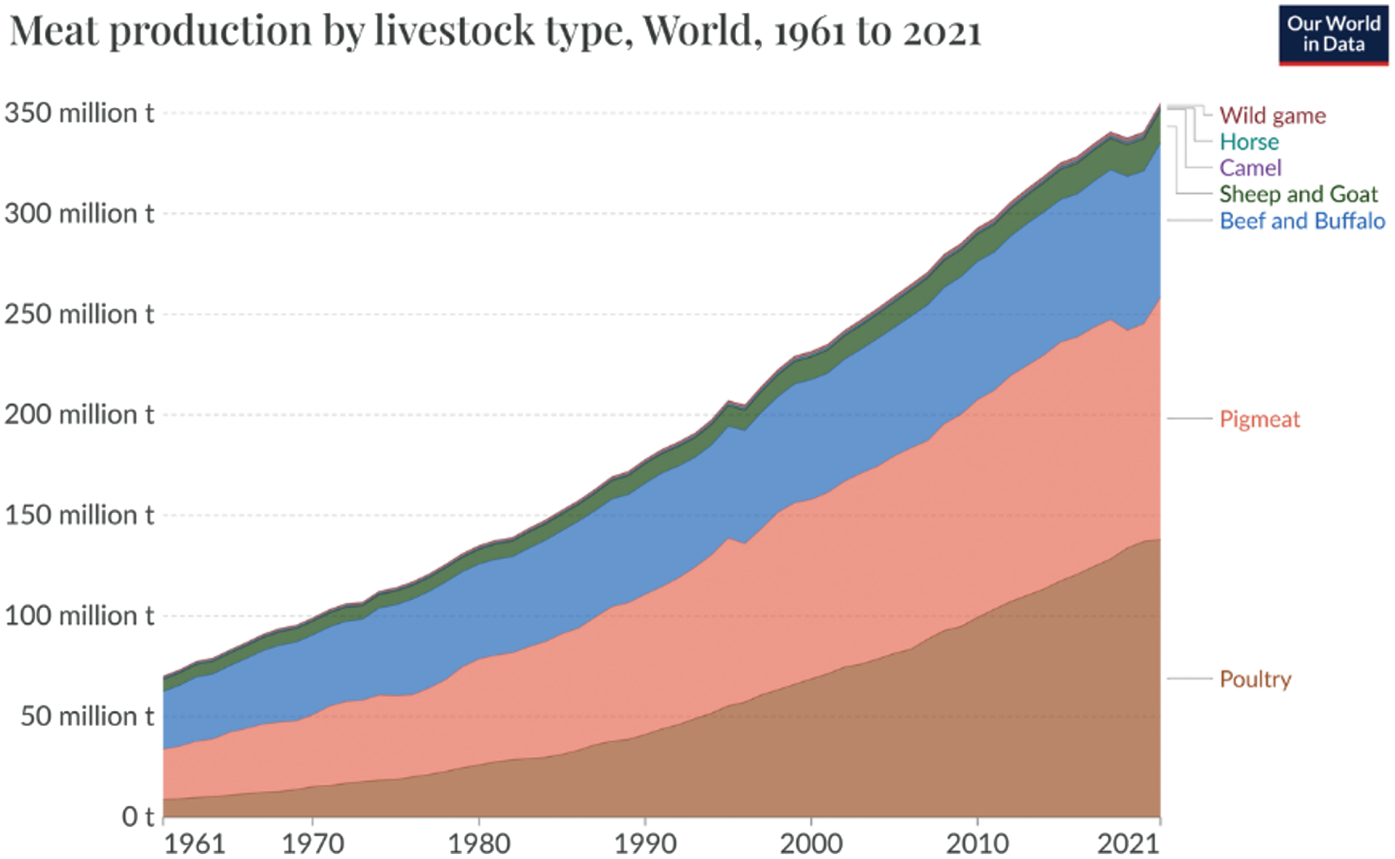
Global meat production by livestock type. Note: Total meat production includes both commercial and farm slaughter. Data provided concern dressed carcass weight, excluding offal and slaughter fats. Source: FAO (https://www.fao.org/faostat/en/#data/QCL). OurWorldInData.org/meat-production • CC BY

The global human population multiplied tenfold between 1700 and 2003, from about 600 million to over 6 billion, reaching more than 8 billion in 2023; from 2050, growth will stabilize to reach around 12 billion in 2100 (United Nations, 2017; Ritchie et al., 2023). The current global human population, combined with the high per capita consumption rate of natural resources, places enormous stress on the sustainability of the planet owing to the demand for water, food, and energy, causing considerable biodiversity losses. Semi-natural grazing areas provide food, support livelihoods for millions of people, and contribute to social and ecological health and well-being (Godde et al., 2020). Animal production is expected to grow, especially in developing countries (FAO, 2018) (Figure 1). Roughly 35% of animal protein worldwide is derived from poultry, 40% from pigs, and 25% from ruminants, mainly cattle and buffalo (Ritchie et al., 2017). To date, livestock production occupies over a quarter of the land surface area of the globe (Robinson et al., 2014).

The global decline in biodiversity is primarily attributed to changes in land use and other human-induced impacts. Within this context, the significance of livestock farming cannot be overlooked, as it contributes to local and global levels of habitat loss, fragmentation, and degradation, which are identified as major drivers of the overall decline in global biodiversity (MEA, 2005). Balancing the needs of production with the imperative of conserving biodiversity remains one of the most critical challenges faced by humanity. The ongoing intensification and expansion of livestock production are expected to amplify interactions between livestock and wildlife, thereby leading to conflicts between production interests and wildlife conservation (Schieltz and Rubenstein, 2016; Gordon, 2018).

This review aims to examine the ecological interplay between livestock and wildlife, evaluate their principal impacts, and pinpoint key factors that facilitate the integration of livestock production with wildlife conservation. Livestock, as defined by Gordon (2018), generally encompasses all domesticated animals reared for productive purposes. Wildlife, as a broader term, encompasses native, non-domesticated organisms, including fungi, plants, and animals (Gordon, 2018). For the purpose of this paper, the term ‘wildlife’ will be restricted to terrestrial vertebrates, namely tetrapods, and “livestock” with a specific emphasis on grazing mammals.

## Livestock Effects on Wildlife

Numerous studies have delved into the impact of livestock on various aspects of the environment, including biodiversity, soil quality, water resources, and climate patterns. The influence of livestock on vegetation and soil dynamics has garnered significant attention in the literature (Asner et al., 2004; Stahlheber and D’Antonio, 2013). Regarding wildlife, the impact of livestock varied, ranging from adverse to favorable, with a predominance of studies highlighting the negative effects (Steinfeld et al., 2006; Gordon, 2018). These effects can manifest directly, such as through competitive interactions, or indirectly, for instance, via alterations in vegetation, facilitation, human presence, and the establishment of cultivated pastures.

In contrast to agricultural and tree plantation practices, extensive grazing methods are often viewed as fostering suitable habitats for wildlife, as they do not entirely disrupt native ecosystems (Steinfeld et al., 2006; FAO, 2009). Nevertheless, this is not universally applicable. For instance, while livestock production in the Río de la Plata Grasslands of temperate South America does not lead to deforestation (Baeza et al., 2022), substantial deforestation is occurring in the tropical regions of South America due to livestock farming and other agricultural activities, leading to significant environmental consequences (Wassenaar et al., 2007).

Habitat loss, fragmentation, and degradation are three of livestock production’s most severe indirect consequences. Globally, Asner et al., (2004) have identified three categories of ecosystem degradation syndromes associated with grazing, one of which is deforestation, which contributes to a substantial loss of biodiversity. Habitat fragmentation can arise from habitat loss and the subdivision of ecosystems due to deforestation. It can also result from the construction of barriers like fencing across different ecosystems. Private land ownership and the use of fences are central features in both intensive and extensive production systems. Fencing, as a management tool, exerts direct and indirect effects on wildlife, at both small and large scales, which can significantly affect species’ movement and migration patterns (e.g., the “dingo” fence in Australia, spanning 5,614 km, as highlighted by Gordon, 2018).

**Figure 2.**
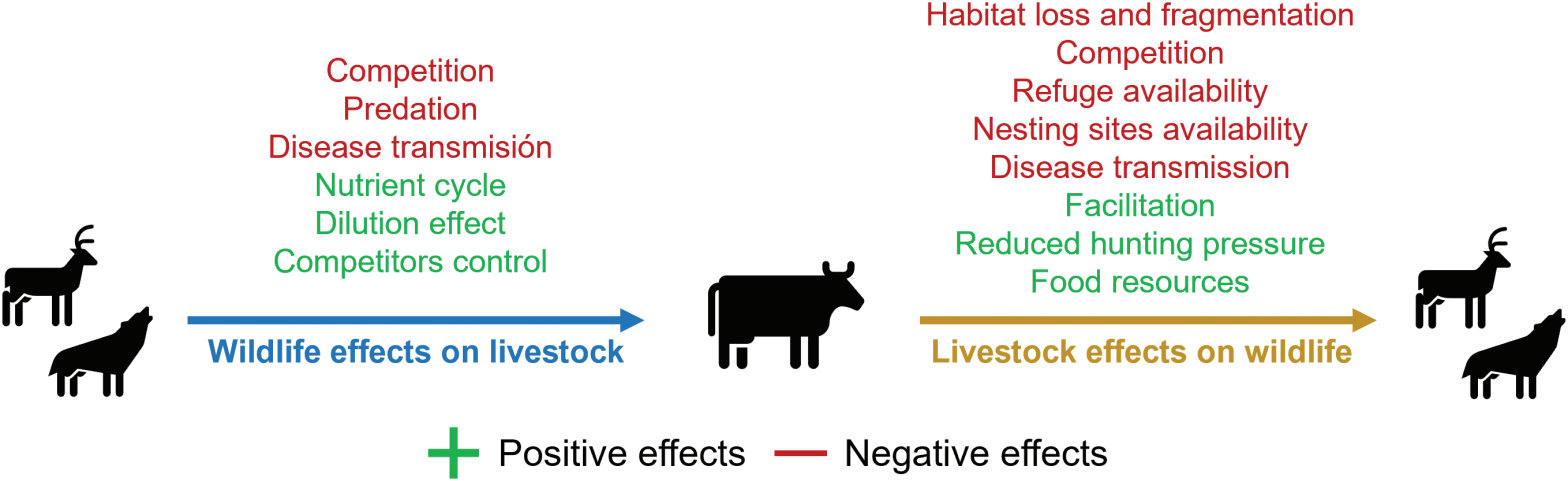
Effects within livestock-wildlife interface. Source: author’s elaboration.

One of the extensively researched effects of livestock is overgrazing (Steinfeld et al., 2006; Schieltz and Rubenstein, 2016), which has reduced the density and biomass of plants and animal species, affected the overall biodiversity and altered the ecological succession, nutrient cycles, and landscape heterogeneity in many regions of the world (Gordon, 2018). However, grazing has also been found to positively affect wildlife: even if it generally reduces forage quantity, it may improve vegetation quality by removing old forage and stimulating new ones. The effect of livestock grazing on native herbivores can, therefore, be negative through direct competition (Schieltz and Rubenstein, 2016); Gordon, 2018; see Box 1 for more details) or positive through facilitation, but can also be positive or negative according to species’ preferences for herbaceous cover and height, as is the case of small mammals, birds, and reptiles (Schieltz and Rubenstein, 2016). Vegetation changes can indirectly influence wildlife by altering prey abundance, diminishing forage quantity and quality, and reducing vegetation refuge and nesting sites (Schieltz and Rubenstein, 2016). This means that the effects vary among wildlife species according to their diet, feeding habits, or even their body size, mainly due to vegetation change (Figure 2).

**Box 1. Livestock and wild herbivores**

Livestock and wild herbivores compete for resources and occupy similar ecological niches (Manzano et al., 2023). Demographic dynamics of livestock and wild herbivores vary between regions and have also changed throughout history.

In Europe, the number of heads of cattle and small ruminants has decreased in recent decades, while other types of livestock have remained stable (FAO, 1997). About wild ungulates, their populations have undergone a notable increase (Massei et al., 2014). In North America, the number of cattle has remained constant in recent decades (FAO, 1997), while populations of wild ruminants have increased (e.g., Rushing et al. 2020). Other factors, such as milder winters, the lack of sufficient predators, reforestation, and an intensification of crop production, might contribute to this pattern (Massei et al., 2014).

There is a downward trend in the populations of small domestic ruminants in most South American countries. However, the number of heads of cattle has increased notably (FAO, 1997). Regarding wild herbivores, some, such as introduced deer and feral pigs, are steadily increasing, while several native herbivores struggle to maintain their numbers (e.g., Relva et al., 2016). Large-scale changes in land use, including deforestation for pasture and crop production, are the main drivers of the observed trends. However, grazing-based livestock systems based on native grasslands can sometimes contribute to biodiversity conservation in this region (de Santiago et al., 2022).

Populations of wild African ungulates are rapidly declining in countries such as Kenya, while livestock, primarily cattle, have increased (Ogutu et al., 2016). However, most species for which comparable long-term data are available have rapidly declined in Kenya, where more considerable changes in land use have occurred, including the disruption of migratory corridors. This suggests that the land use change and the subsequent decline in wildlife observed in East Africa are driven mainly by changes in agricultural policy and land tenure (Homewood et al., 2001).

**Box 2. Livestock-Wildlife Interface in Uruguay**

Uruguay has 11.4 million cattle and 6.6 million sheep, producing 1108 thousand tons of beef, 67 thousand tons of lamb, and 26.6 thousand tons of wool annually (DIEA, 2022). Beef cattle and sheep husbandry are based on extensive grazing in native pastures as the main food source (DIEA, 2022; Zambra et al., 2022).

Regarding the interaction between livestock and mammals, in the recent past, the Pampas deer (*Ozotoceros bezoarticus*) has been one of the most characteristic species in Uruguayan grasslands. Two endemic subspecies currently remain (*O. b. arerunguaensis* in northern Uruguay and *O. b. uruguayensis* in southeast Uruguay), with small and highly isolated populations, mostly on cattle ranches (Cosse et al., 2009). Even though the Uruguayan government has recognized that Pampas deer is a threatened species, declaring the species a living Uruguayan Natural Monument (Ministerial Decree 12/985), no management guidelines have yet been issued, nor any action taken for its effective conservation, which now depends solely on farmers’ management decisions.

In the case of the interaction between livestock and birds, in 2006, and with the encouragement of BirdLife International and its partners in Argentina (Aves Argentinas), Brazil (SAVE Brasil), Paraguay (Guyra Paraguay) and Uruguay (Aves Uruguay), an initiative for the conservation of the grasslands of the Southern Cone of South America was created, which is known as the Grassland Alliance (in Spanish: Alianza del Pastizal). In 2020, the first report concerning birds throughout the Río de la Plata Grasslands indicated that the conservation status of birds is adequate on the properties studied. However, it could be improved through changes in grazing management to achieve higher pastures and more heterogeneous landscapes (Aldabe et al., 2020).

These two cases are related to the presence of livestock in grasslands, but cattle are very common and abundant in native Uruguayan forests, although their effects on forest wildlife and ecosystem functioning have not yet been studied.

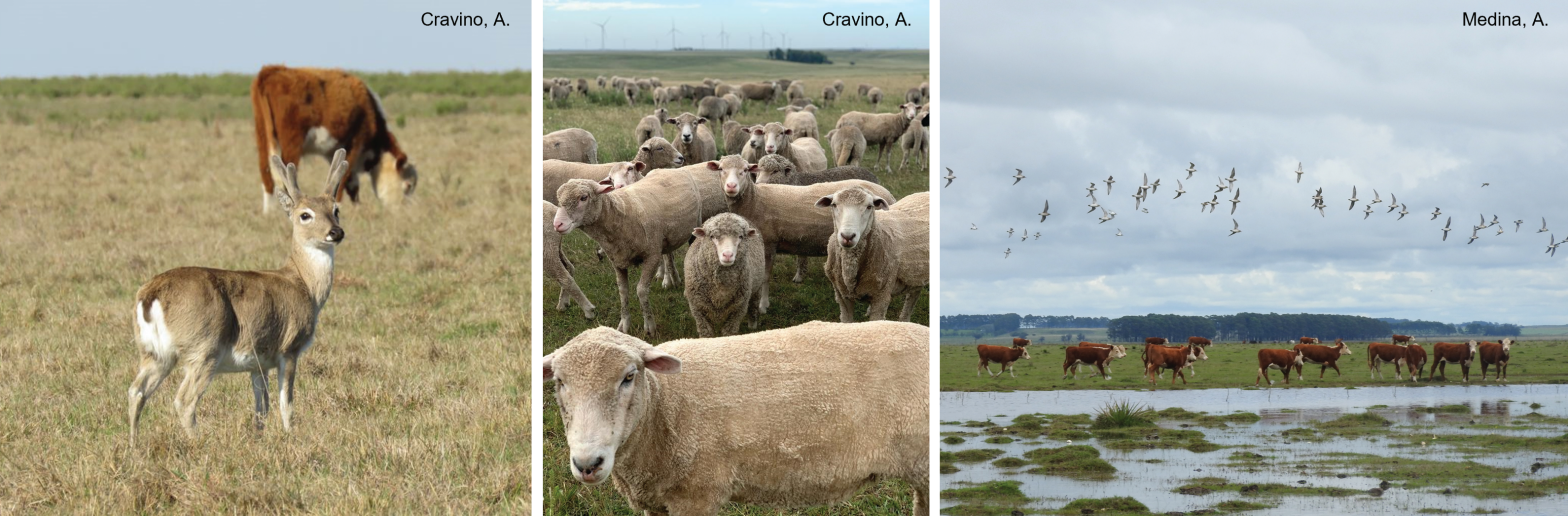

Regarding the presence of livestock itself, some species have clear positive and close associations with livestock owing to the opportunities to feed on insects provided by the livestock, but also to vegetation conditions (Schieltz and Rubenstein, 2016). Domestic animal carcasses may also favor some wild carnivores by increasing the availability of opportunistic feeding. On the other hand, livestock could provide an alternative protein source, diminishing the hunting pressure on wildlife.

## Wildlife Effects on Livestock

When considering the effects of wildlife on livestock, conflicts resulting from negative effects frequently emerge (Gordon, 2018). The most frequently mentioned effects are direct interaction (e.g., predation, disease transmission) or competition for food and water resources (Figure 2). Predation by large carnivores is often perceived as the primary threat in various regions (e.g., Van Eeden et al., 2018), leading to the promotion of eradication campaigns for these carnivores, as in the following examples. This has led to several species’ extinction locally in Europe and North America, including wolves and bears (Thorn et al., 2013; Smith et al., 2014). In Africa and Asia, these tensions have placed constant pressure on lion, cheetah, leopard, and African wild dog populations (Thorn et al., 2013). Similar scenarios are observed in South America with pumas, jaguars (Palmeira et al., 2008), and wild foxes owing to potential sheep predation (Zambra et al., 2022).

Regarding interconnections, predation by large carnivores reduces populations of wild herbivores, thus controlling the risk of disease transmission and allowing the growth of vegetation that will benefit livestock (Pozo et al., 2021). This is particularly important since livestock activities sometimes take place close to protected areas such as national parks and natural water sources due to land use, land cover change, and climate change. This proximity may increase the competition between large wild herbivores and livestock (Manzano et al., 2023).

Finally, when considering wildlife diversity, higher diversity values contribute to maintaining healthy environments (Khalil et al., 2016). The dilution effect hypothesis suggests that healthy and diverse communities limit the spread of certain types of disease (Civitello et al., 2015), signifying that biodiversity losses could worsen epidemics that harm humans and the production of animals, thus emphasizing the need for wildlife-friendly production practices.

## Key Factors: Stocking Rate and Habitat Heterogeneity

The effects of livestock management itself influence wildlife species and need to be considered. As highlighted by Gordon (2018), three distinct approaches exist to strike a balance between the wildlife preservation and livestock production: (1) “fortress conservation” or “land sparing”, in which biodiversity is protected within areas that exclude production, which is intensified outside these areas (Phalan et al., 2011), (2) “land sharing” involving wildlife-friendly farming in the same areas, supporting biodiversity while simultaneously meeting the demands for livestock products (Phalan et al., 2011), and (3) the “win-win” approach where biodiversity is perceived as a provider of ecosystem services (e.g., herbivores control) to the production system, which supports biodiversity outcomes (Gordon et al., 2017). We shall focus on the win-win outcomes.

**Figure 3.**
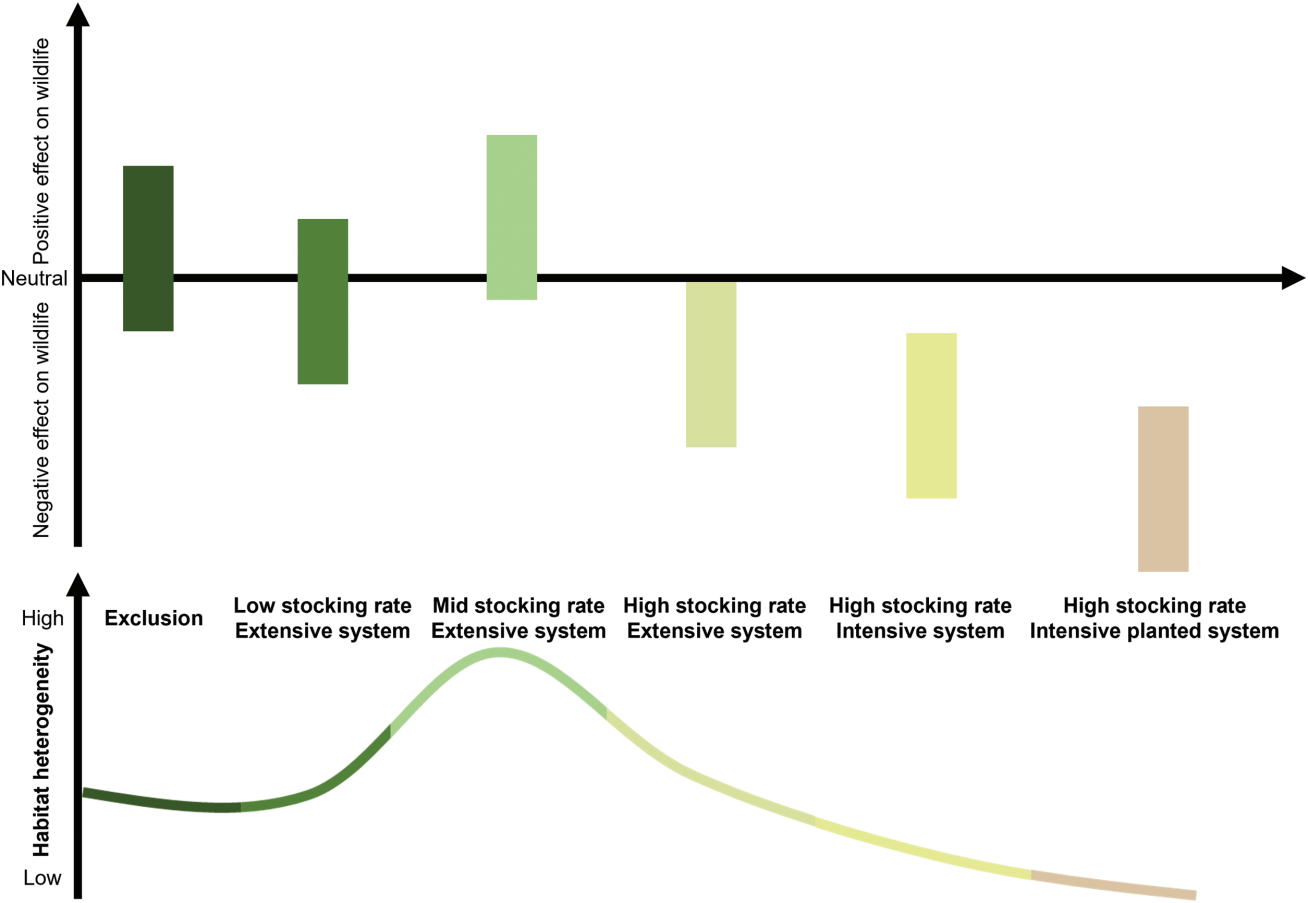
Schematic model representing the variation as regards (a) livestock effects on wildlife (positive and negative) and (b) habitat heterogeneity across a stocking rate gradient. Source: author’s elaboration.

When considering livestock effects as a productive activity, a key factor is stocking rate or livestock grazing intensity. Usually, in livestock production, the management unit is generally a herd (i.e., a group of animals in a paddock or housed together within a facility -Rosa, 2021-) of variable size. A gradient of stocking rate could be drawn according to the management system (e.g., extensive pasture, intensive pasture, and intensive pasture with “pasture improvements”) (Figure 3). Extensive pastures are the dominant practice around the globe, but when the land has high agroecological potential, more intensive pastures occur. However, when the land is scarce, with poor conditions or a bad climate, there is intensive cultivated pasture production (i.e., with associated planted pastures) (Robinson et al., 2011). These may occur at the expense of cropland or through the conversion of forests to pasture.

High grazing pressure, homogenization, and ecosystem degradation inevitably lead to negative repercussions for both wildlife and livestock production (Figure 3), given that extensive livestock systems often depend on native vegetation and water sources as their primary food supply. Typically, when assessing stocking rates, experimental studies have contrasted grazed and exclusion plots, demonstrating that grazing is not an all-or-nothing option, and thus, intermediate conditions must be considered to achieve win-win outcomes (Figure 3).

Ecological theory suggests that local species diversity is maximized when an ecological disturbance is neither too rare nor too frequent, a concept known as the intermediate disturbance hypothesis (Connell, 1978), in which higher diversity is maintained at intermediate levels by maintaining higher habitat heterogeneity. Managed livestock grazing at light to moderate intensities can positively impact rangeland vegetation compared to grazing exclusion or intensive productions (Holechek et al., 2006). Employing low or intermediate stocking rates and implementing rotational herd movements between paddocks tends to foster greater heterogeneity in vegetation structure, driving vegetation dynamics, maintaining biodiversity, and potentially enhancing wildlife habitats (Figure 3; Gordon, 2018). Interspecific competition could also be minimized by increasing habitat heterogeneity. Consequently, win-win solutions have the potential to mitigate adverse effects, while effective management practices could yield positive impacts (Gordon, 2018), thereby averting overgrazing or excessive cattle intensity.

Win-win outcomes have been identified worldwide (see references in Gordon, 2018). Beyond stocking rate management, several options would promote conservation within livestock production systems, such as conservation easements and leases, tax benefits, payments for ecosystem services, wildlife tourism (Gitahi and Fitzgerald, 2011), or developing distinctive brands that reward good livestock practices.

## Conclusions

The expansion of livestock production is expected to continue in tandem with human population growth and the demand for food. This expansion significantly impacts wildlife, particularly when natural ecosystems are supplanted by artificial pasturelands for grazing, as observed in subtropical and tropical forests across South America. Through habitat loss and fragmentation, livestock production indirectly engenders notable adverse consequences for wildlife in various regions globally.

When livestock production occurs in native grasslands, adverse effects on wildlife are more frequent, but some species can also be favored. The extent and strength of these effects are related to the stocking rate and the habitat heterogeneity in livestock management practices. Nevertheless, further research on grazing conditions falling within the intermediate range is imperative.

Extensive systems that incorporate moderate stocking rates and appropriate herd rotation to uphold habitat heterogeneity can foster a more appropriate equilibrium between production and biodiversity conservation. Such management strategies are likely to yield win-win outcomes by promoting vegetation heterogeneity and providing opportunities for the conservation of wildlife species, consequently benefiting livestock production. Effective dialogue between stakeholders, farmers, and scientists is pivotal in achieving sustainability and balancing wildlife conservation and livestock production.

## About the Authors

**Alexandra Cravino** holds a Ph.D. in Biological Sciences from the Program for the Development of Basic Sciences (PEDECIBA) from the Faculty of Sciences (FCien) at the University of the Republic in Montevideo, Uruguay. She is an Assistant Researcher in the Biodiversity and Conservation Ecology Group at the Institute of Ecology and Environmental Sciences (FCien-UdelaR). She is a member of the National System of Researchers (SNI). Her research interests encompass wildlife in protected areas, the effects of afforestation and livestock on mammals, and invasive exotic species monitoring.

**Alberto Perelló** is a Spanish veterinarian at SabioTec Spin-off S.L. where he works on animal tuberculosis diagnosis and biosafety assessment in extensive livestock farms. At the same time, after graduating in Veterinary Medicine in León in 2022, he started his career at IREC (*Instituto de Investigación en Recursos Cinegéticos*) in Ciudad Real (Spain) working on health surveillance and biodiversity monitoring at the wildlife-livestock interface to characterize pathogens and reservoir communities, all this encompassed in the “conservation medicine” concept.

**Alejandro Brazeiro** is a professor and researcher in the Biodiversity and Conservation Ecology Group at the Institute of Ecology and Environmental Sciences from the Faculty of Sciences (FCien) at the University of the Republic (Montevideo, Uruguay). He specializes in Biodiversity and Conservation Ecology, with a particular focus on native forests. Additionally, he is a member of the National System of Researchers (SNI). As an accomplished academic, he has authored a hundred published works, which include articles, book chapters, and technical reports.

## Credit

AC: Conceptualization, Investigation, Writing—original draft; AP: Conceptualization, Investigation, Writing—review & editing; AB: Conceptualization, Investigation, Writing—review & editing.

## References

[CIT0001] Aldabe, J., G. Bencke, P. Grilli, A. Medina, and L. Sforza. 2020. Evaluación de estado de las aves en predios de la Alianza del Pastizal. Guyra Paraguay & SAVE Brasil: Alianza del Pastizal: Aves Argentinas, Aves Uruguay.

[CIT0041] Asner GP , Elmore AJ, Olander LP, Martin RE, Harris T. 2004. Grazing systems, ecosystem responses, and global change. Ann. Rev. Environ. Res. 29:261–299. 10.1146/annurev.energy.29.062403.102142

[CIT0002] Baeza S , Vélez-Martin E, De Abelleyra D, Banchero S, Gallego F, Schirmbeck J, Veron S, Vallejos M, Weber E, Oyarzabal M, et al. 2022. Two decades of land cover mapping in the Río de la Plata grassland region: the mapbiomas pampa initiative. Remote Sensing Applications: Society and Environment 28:100834. 10.1016/j.rsase.2022.100834

[CIT0003] Civitello DJ , Cohen J, Fatima H, Halstead NT, Liriano J, McMahon TA, Ortega CN, Sauer EL, Sehgal T, Young S, et al. 2015. Biodiversity inhibits parasites: broad evidence for the dilution effect. Proc. Natl. Acad. Sci. USA. 112(28):8667–8671. 10.1073/pnas.1506279112 26069208 PMC4507196

[CIT0004] Connell JH. 1978. Diversity in tropical rain forests and coral reefs. Science. 199(4335):1302–1310. 10.1126/science.199.4335.1302 17840770

[CIT0005] Cosse M , González S, Giménez-Dixon M. 2009. Feeding ecology of *Ozotoceros bezoarticus*: conservation implications in Uruguay. Iheringia. Série. Zoologia. 99(2):158–164. 10.1590/s0073-47212009000200007

[CIT0006] de Santiago MF , Barrios M, D’Anatro A, García LF, Mailhos A, Pompozzi G, Rehermann S, Simó M, Tesitore G, Teixeira de Mello F, et al. 2022. From theory to practice: can LEAP/FAO biodiversity assessment guidelines be a useful tool for knowing the environmental status of livestock systems? Sustainability. 14(23):16259. 10.3390/su142316259

[CIT0007] DIEA, 2022. Anuario Estadístico Agropecuario. Ministerio de Ganadería Agricultura y Pesca. Montevideo: Gráfica Mosca.

[CIT0008] FAO. 1997. FAOSTAT Statistical Database. Rome: Food and Agriculture Organization.

[CIT0009] FAO. 2009. Sustaining Communities, Livestock and Wildlife: A Guide to Participatory Land-Use Planning. Rome: Food and Agriculture Organization.

[CIT0010] FAO. 2018. Shaping the future of livestock sustainably, responsibly, efficiently. Berlin: The 10th Global Forum for Food and Agriculture.

[CIT0011] Gitahi, N. and K. H. Fitzgerald. 2011. Conserving wildlife on private lands: the legal framework for landownership and new tools for land conservation. In: Georgiadis N.J., editors. Conserving wildlife in African landscapes: Kenya’s Ewaso Ecosystem. Smithsonian Contributions to Zoology 632. Washington, DC, USA: Smithsonian Institution Scholarly Press.

[CIT0012] Godde CM , Boone RB, Ash AJ, Waha K, Sloat LL, Thornton PK, Herrero M. 2020. Global rangeland production systems and livelihoods at threat under climate change and variability. Environmental Research Letters 15(4):044021. 10.1088/1748-9326/ab7395

[CIT0013] Gordon IJ. 2018. Review: Livestock production increasingly influences wildlife across the globe. Animal. 12(s2):s372–s382. 10.1017/S1751731118001349 30109828

[CIT0014] Gordon, I.J., G.R. Squire, and H.H.T. Prins. 2017. Conclusion: re-engaging agriculture with nature. In: Gordon I.J., Prins H.H.T., Squire G.R., editors. Food production and nature conservation: conflicts and solutions. Routledge, London, UK: Earthscan Food and Agriculture.

[CIT0015] Holechek JL , Baker TT, Boren JC, Galt D. 2006. Grazing impacts on rangeland vegetation: what we have learned. Rangelands. 28(1):7–13. 10.2111/1551-501x(2006)28.1[7:giorvw]2.0.co;2

[CIT0016] Homewood K , Lambin EF, Coast E, Kariuki A, Kikula I, Kivelia J, Said M, Serneels S, Thompson M. 2001. Long-term changes in Serengeti-Mara wildebeest and land cover: pastoralism, population, or policies? Proceedings of the National Academy of Sciences of the United States of America 98(22):12544–12549. 10.1073/pnas.221053998 11675492 PMC60090

[CIT0017] Khalil H , Ecke F, Evander M, Magnusson M, Hörnfeldt B. 2016. Declining ecosystem health and the dilution effect. Scientific Reports 6:31314. 10.1038/srep31314 27499001 PMC4976314

[CIT0018] Manzano P , del Prado A, Pardo G.2023. Comparable GHG emissions from animals in wildlife and livestock-dominated savannas. NPJ Clim. Atmos. Sci. 6(1):27. 10.1038/s41612-023-00349-8

[CIT0019] Massei G , Kindberg J, Licoppe A, Gačić D, Šprem N, Kamler J, Baubet E, Hohmann U, Monaco A, Ozoliņš J, et al. 2014. Wild boar populations up, numbers of hunters down? A review of trends and implications for Europe. Pest Manag. Sci. 71(4):492–500. 10.1002/ps.3965 25512181

[CIT0020] MEA 2005. Ecosystems and human well-being: Biodiversity synthesis. Washington DC, US: World Resources Institute.

[CIT0021] Ogutu JO , Piepho H-P, Said MY, Ojwang GO, Njino LW, Kifugo SC, Wargute PW. 2016. Extreme wildlife declines and concurrent increase in livestock numbers in Kenya: what are the causes? PLoS One 11(9):e0163249. 10.1371/journal.pone.0163249 27676077 PMC5039022

[CIT0022] Palmeira FBL , Crawshaw PG, Haddad CM, Ferraz KMPMB, Verdade LM. 2008. Cattle depredation by puma (*Puma concolor*) and jaguar (*Panthera onca*) in central-western Brazil. Biol. Conserv. 141:118–125. 10.1016/j.biocon.2007.09.015

[CIT0023] Phalan B , Onial M, Balmford A, Green RE. 2011. Reconciling food production and biodiversity conservation: land sharing and land sparing compared. Science. 333(6047):1289–1291. 10.1126/science.1208742 21885781

[CIT0024] Pozo RA , Cusack JJ, Acebes P, Malo JE, Traba J, Iranzo EC, Morris-Trainor Z, Minderman J, Bunnefeld N, Radic-Schilling S, et al. 2021. Reconciling livestock production and wild herbivore conservation: challenges and opportunities. Trends Ecol. Evol. 36(8):750–761. 10.1016/j.tree.2021.05.002 34103191

[CIT0025] Relva MA , Sanguinetti G. 2016. Ecología, impacto y manejo del ciervo colorado (*Cervus elaphus*) en el noroeste de la Patagonia, Argentina. Mastozool. neotrop 23(2):221–238.

[CIT0027] Ritchie, H., et al. 2023. Population Growth. (Accessed July 2020). https://ourworldindata.org/population-growth.

[CIT0028] Ritchie, H., P. Rosado, and M. Roser. 2017. Meat and Dairy Production. (Accessed July 2020). https://ourworldindata.org/meat-production.

[CIT0029] Robinson, T.P., et al. 2011. Global livestock production systems. Rome: Food and Agriculture Organization and International Livestock Research Institute (ILRI).

[CIT0030] Robinson TP , Wint GRW, Conchedda G, Van Boeckel TP, Ercoli V, Palamara E, Cinardi G, D'Aietti L, Hay SI, Gilbert M. 2014. Mapping the global distribution of livestock. PLoS One 9(5):e96084. 10.1371/journal.pone.0096084 24875496 PMC4038494

[CIT0031] Rosa GJM. 2021. Grand challenge in precision livestock farming front. Anim. Sci. 2. 10.3389/fanim.2021.650324

[CIT0032] Rushing CS , Rohrbaugh RW, Fiss CJ, Rosenberry CS, Rodewald AD, Larkin JL. 2020. Long-term variation in white-tailed deer abundance shapes landscape-scale population dynamics of forest-breeding birds. For. Ecol. Manag. 456:117629. 10.1016/j.foreco.2019.117629

[CIT0040] Schieltz JM , Rubenstein DI. 2013. Evidence based review: positive versus negative effects of livestock grazing on wildlife. What do we really know? Environ. Res. Lett. 11:113003. 10.1088/1748-9326/11/11/113003

[CIT0033] Stahlheber KA , D’Antonio CM. 2013. Using livestock to manage plant composition: a meta-analysis of grazing in California Mediterranean grasslands. Biol. Conserv. 157:300–308. 10.1016/j.biocon.2012.09.008

[CIT0034] Steinfeld, H., P. Gerber, T. Wassenaar, V. Castel, M. Rosales, and C. de Haan. 2006. Livestock’s long shadow. The livestock, environment and development initiative (LEAD). Rome: Food and Agriculture Organization.

[CIT0042] Thorn M , et al. 2013. Characteristics and determinants of human-carnivore conflict in South African farmland. Biodivers. Conserv. 22:1715–1730. 10.1007/s10531-013-0508-2

[CIT0035] United Nations. 2017. World population prospects: The 2017 revision, key findings and advance tables. working paper No. ESA/P/WP/248. Population Division: Department of Economic and Social Affairs.

[CIT0036] Van Eeden LM , Crowther MS, Dickman CR, Macdonald DW, Ripple WJ, Ritchie EG, Newsome TM. 2018. Managing conflict between large carnivores and livestock. Conserv. Biol. J. Soc. 32(1):26–34. 10.1111/cobi.12959 28556528

[CIT0037] Wassenaar T , Gerber P, Verburg PH, Rosales M, Ibrahim M, Steinfeld H. 2007. Projecting land use changes in the Neotropics: the geography of pasture expansion into the forest. Glob. Environ. Change. 17(1):86–104. 10.1016/j.gloenvcha.2006.03.007

[CIT0039] Zambra N , Piaggio J, Ungerfeld R. 2022. Characteristics of sheep farms and livestock practices that influence sheep predation in Uruguay. Mastozool. Neotrop. 29(1):e0569. 10.31687/saremMN.22.29.1.05.e0569.

